# General Grant's Disease

**Published:** 1885-03

**Authors:** 


GEN. GRANTS DISEASE.—The *Medical Record* of the 24th. of April last gives the result of an examination as follows:

"The microscopic examination of the specimen removed from Gen. Grant's throat has declared the disease to be epithelioma. But epithelioma, barring accidents from complications, is not a very rapidly progressive disease. It may continue for months, slowly eating its way into surrounding tissues without of itself killing the patient. The progress of the throat trouble, under well-recognized methods of treatment, has thus far not been such as would lead physicians to believe that the microscope was in error in confirming the original diagnosis."

Prof. Frank Abbott, M. D. of New York City, in an article published in the *Independent Practitioner*, April No. describes the condition of Gen. Grant's mouth on the 8th, of November 1884, from an examinotion made by him at the request of Dr. Fordyce Baker. The right ripper first molar was found to be dead, with an abscess at the apex of the anterior buccal root, it was considerably projected from its original position, and the neck and one half the length of the roots were covered with tartar. This tooth was removed, and the relief from pain was highly satisfactory. An examination made by Dr. Abbott on the 14th, of the same month showed the second and third right superior molars to be projecting from a quarter to three eights of an inch, the necks and two-thirds of the length of their roots being thickly coated with tartar, of a dark brown or black variety, the sockets being almost entirely absorbed, while the teeth were very loose. The second molar was very badly decayed, the side wall broken down, and the pulp dead. The surrounding gum was very much irritated, the inflammation extending to the roof of the mouth and back into the fauces. These teeth were removed and the tartar cleaned from the other teeth in the mouth. Dr. Abbott also says that the side of the tongue had been constantly rubbing against the rough and ragged surfaces of the broken tooth and the tartar incrustations, and the inflammation had extended into the vault and down that side of the patient's throat, and he very reasonably concludes that "here may have been a factor, at least, in the localization of the painful disease from which the General is now suffering, instead of attributing, it altogether to the habit of smoking, which seems to be the prevailing opinion, at least among the laity."

An examination with an illumating apparatus on the 25th, of April last, is described as follows in the New York papers.

"The state of the General's throat being such that he could not open his mouth wide enough to admit of the old instrument, the new one was put into operation for the first time in this country. It consists of a dynamo generator, worked by a crank and a tongue depressor and incandescent lamp in one. The view of the General's throat under the new light revealed a very discouraging state of affairs. The gums are honeycombed and for a distance down the uvula presented the appearance of a red rough surface covered with mucus. To to this is attributed the pain and uneasiness which the General has been suffering from for the last few days. It was the opinion that the doctors have made no mistake in their diagnosis and that the General's case is a hopeless one, and that the General's was such a critical case that no definite opinion can be given of it."

---

